# Multi-Center Outcome Analysis of 16 Face Transplantations – A Retrospective OPTN Study

**DOI:** 10.3389/ti.2025.14107

**Published:** 2025-01-29

**Authors:** Leonard Knoedler, Thomas Schaschinger, Tobias Niederegger, Gabriel Hundeshagen, Adriana C. Panayi, Curtis L. Cetrulo, Maxime Jeljeli, Elena Hofmann, Max Heiland, Steffen Koerdt, Alexandre G. Lellouch

**Affiliations:** ^1^ Department of Oral and Maxillofacial Surgery, Charité - Universitätsmedizin Berlin, Corporate Member of Freie Universität Berlin, Humboldt-Universität zu Berlin, and Berlin Institute of Health, Berlin, Germany; ^2^ Department of Hand, Plastic and Reconstructive Surgery, Burn Center, BG Trauma Hospital Ludwigshafen, University of Heidelberg, Ludwigshafen, Germany; ^3^ Department of Plastic and Hand Surgery, Burn Center, BG Trauma Hospital Ludwigshafen, University of Heidelberg, Ludwigshafen, Germany; ^4^ Vascularized Composite Allotransplantation Laboratory, Massachusetts General Hospital, Harvard Medical School, Boston, MA, United States; ^5^ Shriners Children’s Boston, Boston, MA, United States; ^6^ Cedars-Sinai Medical Center, Los Angeles, CA, United States

**Keywords:** face transplantation, facial transplantation, vascularized composite allotransplantation, VCA, OPTN

## Abstract

Facial Vascularized Composite Allotransplantation (fVCA) restores form and function for patients with severe facial disfigurements, yet multi-center outcome data remain scarce. We accessed the Organ Procurement and Transplantation Network (OPTN) database from 2008 to 2024 to identify all full- or partial-face fVCA recipients, excluding patients under 18 years and those with physiologically impossible BMIs. Of 25 identified patients, 16 (64%) met inclusion criteria (69% male; mean age 43 ± 14 years). Recipients experienced a median of 5 [IQR 0.0–10] acute rejection episodes, which correlated with inotrope use during donor procurement (p = 0.033). On average, patients were hospitalized 2.4 ± 1.8 times, with arginine vasopressin (AVP) administration linked to fewer hospitalizations (p = 0.035). Seven recipients (44%) experienced complications, and extended-criteria donor (ECD) status was associated with higher complication rates (p = 0.049). These findings underscore the promise of fVCA to address complex facial defects while identifying key risk factors—particularly inotrope use and ECD status, while AVP administration may mitigate hospital stays. Further studies with larger cohorts are warranted to refine perioperative strategies, improve outcomes, and expand the clinical utility of fVCA.

## Graphical Abstract

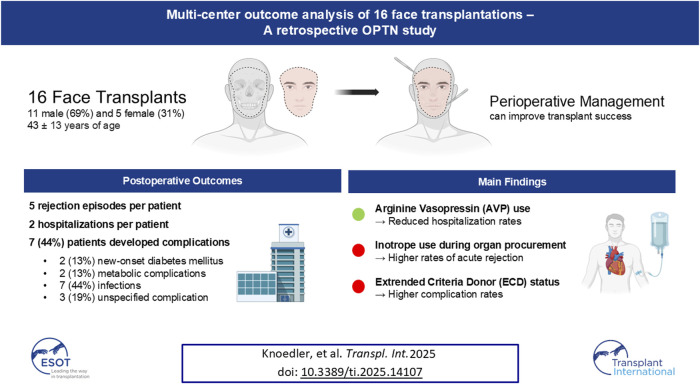

## Introduction

Facial Vascularized Composite Allotransplantation (fVCA) has expanded the reconstructive ladder, providing a novel strategy for patients with extended facial defects. Such defects include severe burns or traumatic accidents. Conventional reconstructive techniques (e.g., full-thickness skin grafts, local flap surgery) may provide insufficient wound coverage and healing in these scenarios [1–3]. First performed in 2005, fVCA has since evolved from an experimental procedure to a robust reconstructive route, offering a lifeline to patients with severe facial disfigurements. Besides improving basic functions (e.g., breathing, eating, speaking), fVCA can provide a renewed sense of personal identity and social integrity [4].

Currently, more than 50 fVCA have been performed worldwide [5]. The main barriers to increasing the number of fVCA procedures and broadening the access to fVCA care include the need for life-long immunosuppression, immune rejection, which has led to two cases of re-transplantation, and the paucity of multi-center and multi-surgeon fVCA outcome research [6]. Over the past decade, promising pathways to induce immunotolerance and reduce/taper immunosuppressive drugs have been proposed [7, 8]. Similarly, recent research has focused on defining acute and chronic rejections after fVCA surgery, as well as investigating novel diagnostic techniques to promptly detect rejection episodes [9].

Despite such advancements, fVCA outcomes can vary widely [10–12]. This variability is multifactorial, and includes patient health, extent of fVCA, and comorbid conditions. Therefore, understanding risk factors predisposing to poorer surgical outcomes and transplant survival is crucial for tailoring preoperative counseling, perioperative protocols, and postoperative monitoring. However, to date, there is a scarcity of comprehensive studies investigating the outcomes and risk factors of fVCA surgery. While these studies may provide valuable insights into single-surgeon and/or single-center center experiences they often lack the generalizability needed to inform decision-making in a broader context.

In contrast, the use of multi-institutional and multi-surgeon databases such as the Organ Procurement and Transplantation Network (OPTN) database can overcome these limitations. The OPTN database provides a dataset that records a wide range of information across the transplantation process. Capturing more than 100,000 candidates for solid organ transplantation, the OPTN dataset also records information on VCA surgery. This multi-faceted patient population may provide a more generalizable reflection of real-world clinical practices. To date, the OPTN database has only been accessed for descriptive research work on VCA surgery [13].

In this study, we queried the OPTN database to investigate outcomes after fVCA surgery and identify risk factors for adverse events. For fVCA providers, these insights can help advance the preoperative patient screening and perioperative treatment algorithms. Identifying high-risk patients may reduce postoperative morbidity and prolong graft survival. On the other hand, these lines of research may empower patients with knowledge about potential risks and benefits following fVCA surgery. Thus, patients may participate more actively in their care decisions, leading to more personalized and satisfactory healthcare experiences. Ultimately, the herein presented data can navigate the development of best-practice guidelines and protocols and improve the quality and safety of surgical fVCA care.

## Patients and Methods

### Data Source and Patient Selection

Data were obtained from the OPTN database, which was developed by the United States Organ Donation and Transplant System (UNOS). This comprehensive database contains detailed records of every organ donation and transplant event in the United States. A search of the OPTN database was conducted to identify all transplant cases involving fVCAs dating back to 10 December 2008. From an initial cohort of 172 entries, 25 cases involving either isolated or combined fVCA transplants were identified. Nine patients were excluded due to missing follow-up data on outcome measurements, resulting in a final cohort of 16 fVCA transplant cases eligible for outcome analysis.

### Variable Extraction

fVCA recipient and donor demographics, transplant and operative data were extracted for analysis. Transplant recipient data were evaluated as follows: (a) recipient demographics [gender, age, ethnicity, primary diagnosis, body mass index (BMI), weight, height, Calculated Panel Reactive Antibody (CPRA) score], (b) laboratory values (serum creatinine, hemoglobin A1c), (c) immunological characteristics [AB0 classification, donor-recipient AB0 mismatch level, Human Leukocyte Antigen (HLA) mismatch level, A locus mismatch level, B locus mismatch level, DR locus mismatch level, computed donor antigens, HLA A1 antigen, HLA A2 antigen, HLA B1 antigen, HLA B2 antigen, HLA DR1 antigen, HLA DR2 antigen, HBV core antibody, HBV surface antigen, HCV serostatus, EBV serostatus, CMV status], and (d) evaluated scores [Physical Functioning (PF) score, Role-Physical (RP) score, Bodily Pain (BP) score, General Health (GH) score, Vitality (VT) score, Social Functioning (SF) score, Role-Emotional (RE) score, Mental Health (MH) score].

We investigated the following donor data: (a) donor demographics [gender, age, ethnicity, BMI, weight, height, type, Expanded Criteria Donor (ECD) status], and (b) immunological characteristics [AB0 classification, HBV core antibody, HBV surface antigen, HBV NAT test result, HCV antibody, HCV NAT test result, EBV (VCA) (IgG) status, EBV (VCA) (IgM) status, risk for blood-borne disease transmission].

With regards to transplant characteristics and perioperative data, we evaluated previous transplants of the same organ, instances of multiple VCA transplantations, use of additional allografts, warm ischemia time, cold ischemia time, distance of donor hospital to transplant center, use of inotropic medication during donor organ procurement, donor administration of arginine vasopressin (AVP) within 24 h pre-cross clamp, donor administration of insulin within 24 h pre-cross clamp, protein in donor urine, recipient pre-transplant blood transfusion, recipient coagulopathies, recipient pre-transplant life support, recipient other risk factors, recipient use of tolerance induction technique, skin type, UNOS transplant region, UNOS listing region, transplant allocation type, and year of transplant.

The postoperative outcomes investigated included the number of acute rejection episodes, the number of hospitalizations, and the occurrence of any complication. The number of acute rejection episodes was evaluated for each patient by checking for the occurrence at each follow-up stamp. Any complication was defined as the occurrence of at least one of the following events within the entire follow-up period: new-onset diabetes mellitus, metabolic complication, infectious complication, or other complication.

### Statistical Analysis

Data were collected and securely stored using an electronic laboratory notebook (LabArchives, LLC, San Marcos, CA, United States). Analyses were conducted using GraphPad Prism (V10 for MacOS, GraphPad Software, La Jolla, CA, United States) and Python within the Google Colaboratory environment (Google Colab). Spearman’s rank correlation was employed to assess relationships between continuous variables, such as age and BMI. For categorical variables, including recipient AB0 blood group and UNOS transplant region, the Kruskal-Wallis test was applied. In instances where statistical significance was found, post-hoc pairwise comparisons were carried out using Dunn’s test with Bonferroni correction to determine specific group differences. A significance threshold of p < 0.05 was used for all tests.

## Results

### Transplant Recipient Demographics

The studied cohort consisted of 16 patients who underwent fVCA surgery. The average age at the time of transplantation was 43 ± 14 years. Most patients were male (n = 11; 69%) and of white ethnicity (n = 7; 44%). At the time of surgery, the mean BMI was 26 ± 7.5 kg/m^2^. The primary cause for fVCA was trauma (n = 9; 56%; Table 1).

**TABLE 1:** Recipient demographics, medical, and immunological information. Reported as n (%), unless otherwise stated.

Demographics	Recipients (n = 16)	
Gender		
Female	5	(31)
Male	11	(68)
Age [years], mean ± SD		
At transplantation	43	±14
At listing	43	±16
Ethnicity		
White, Non-Hispanic	7	(44)
Black, Non-Hispanic	1	(6.2)
Unknown	8	(50)
Primary diagnosis		
Trauma	9	(56)
Burn/explosion	6	(38)
Unknown	1	(6)
Survival time [days], mean ± SD	1,764	±1,226
BMI [kg/m^2^], mean ± SD		
At transplantation	26	±7.5
At registration	25	±8.4
Weight [kg], mean ± SD		
At transplantation	78	±30
At listing	73	±29
At registration	73	±29
Height [cm], mean ± SD		
At transplantation	171	±10
At registration	152	±56
CPRA score [%], median (IQR)		
At transplantation	23	(0–30)
At listing	30	(20–38)
Laboratory values		
Serum creatinine [mg/dL], mean ± SD		
Pre-transplant	0.84	±0.25
Discharge	0.86	±0.23
Hemoglobin A1c (%), median (IQR)		
Pre-transplant	0.0	(0.0–5.0)
Discharge	0.0	(0.0–6.0)
Immunological Characteristics		
AB0		
0	5	(31)
A	4	(25)
Unknown	7	(44)
Donor-recipient ABO match level		
1	8	(50)
2	1	(6.2)
Unknown	7	(44)
HLA mismatch level		
1	1	(6.2)
4	3	(19)
5	2	(13)
6	2	(13)
Unknown	8	(50)
A locus mismatch level		
0	2	(13)
1	3	(19)
2	3	(19)
Unknown	8	(50)
B locus mismatch level		
0	1	(6.2)
2	7	(44)
Unknown	8	(50)
DR locus mismatch level		
1	4	(25)
2	4	(25)
Unknown	8	(50)
Computed donor antigens, median (IQR)		
A1	2	(1.0–5.0)
A2	28	(3.0–31)
B1	11	(7.8–39)
B2	45	(25–61)
DR1	7	(4.0–8.5)
DR2	15	(13–17)
HLA A1 antigen		
1	3	(19)
2	3	(19)
11	1	(6.2)
32	1	(6.2)
Unknown	8	(50)
HLA A2 antigen		
2	1	(6.2)
3	1	(6.2)
11	2	(13)
24	1	(6.2)
30	1	(6.2)
31	1	(6.2)
Unknown	9	(56)
HLA B1 antigen		
7	2	(13)
8	1	(6.2)
35	1	(6.2)
51	1	(6.2)
53	1	(6.2)
60	1	(6.2)
63	1	(6.2)
Unknown	8	(50)
HLA B2 antigen		
8	1	(6.2)
27	1	(6.2)
41	2	(13)
44	1	(6.2)
57	1	(6.2)
58	1	(6.2)
Unknown	9	(56)
HLA DR1 antigen		
4	2	(13)
7	1	(6.2)
11	1	(6.2)
13	2	(13)
15	1	(6.2)
17	1	(6.2)
Unknown	8	(50)
HLA DR2 antigen		
4	2	(13)
11	1	(6.2)
13	1	(6.2)
15	1	(6.2)
17	2	(13)
Unknown	9	(56)
HBV core antibody		
Positive	1	(6.2)
Negative	13	(81)
Unknown	2	(13)
HBV surface antigen		
Positive	1	(6.2)
Negative	13	(81)
Unknown	2	(13)
HCV serostatus		
Positive	1	(6.2)
Negative	14	(88)
Unknown	1	(6.2)
EBV serostatus		
Positive	14	(88)
Negative	1	(6.2)
Unknown	1	(6.2)
CMV status		
Positive	9	(56)
Negative	6	(38)
Unknown	1	(6.2)
Evaluated Scores		
Physical Functioning (PF) score [0–100 scale], mean ± SD	70	±35
Role-Physical (RP) score [0–100 scale], mean ± SD	57	±39
Bodily Pain (BP) score [0–100 scale], mean ± SD	67	±31
General Health (GH) score [0–100 scale], mean ± SD	85	±11
Vitality (VT) score [0–100 scale], mean ± SD	67	±27
Social Functioning (SF) score [0–100 scale], mean ± SD	57	±39
Role-Emotional (RE) score [0–100 scale], mean ± SD	87	±17
Mental Health (MH) score [0–100 scale], mean ± SD	87	±14

CPRA score, Calculated Panel Reactive Antibody score.

Serum creatinine levels rose from a pre-transplant average of 0.84 ± 0.25 mg/dL to 0.86 ± 0.23 mg/dL at discharge. The mean postoperative survival duration was 1,764 ± 1,226 days. Performance on the GH score resulted in an average of 85 ± 11 points on an 100 points scale (Table 1).

### Transplant Donor Demographics

The 16 donors were mostly male (n = 11; 69%) and of white ethnicity (n = 15; 94%) with a mean age of 38 ± 14 years and a BMI of 27 ± 4.0 kg/m^2^. Most donors (n = 12; 75%) were not classified as ECDs. The most common AB0 group was type 0 (n = 9; 56%; Table 2).

**TABLE 2:** Donor demographics and immunological information. Reported as n (%), unless otherwise stated.

Demographics	Donors (n = 16)	
Gender		
Female	5	(31)
Male	11	(69)
Age [years], mean ± SD	38	±14
Ethnicity		
White, Non-Hispanic	15	(94)
Black, Non-Hispanic	1	(6.3)
BMI [kg/m^2^], mean ± SD	27	±4.0
Weight [kg], mean ± SD	78	±16
Height [cm], mean ± SD	171	±10
ECD		
Yes	4	(25)
No	12	(75)
Immunological Characteristics		
Donor AB0		
0	9	(56)
A	2	(13)
A1	5	(31)
HBV core antibody		
Negative	16	(100)
HBV surface antigen		
Negative	16	(100)
HBV NAT test result		
Negative	6	(38)
Unknown	10	(62)
HCV antibody		
Unknown	16	(100)
HCV NAT test result		
Negative	6	(38)
Unknown	10	(62)
HIV NAT test result		
Negative	6	(38)
Unknown	10	(62)
EBV (VCA) (IgG) status		
Positive	14	(88)
Negative	2	(13)
EBV (VCA) (IgM) status		
Negative	16	(100)
Risk for blood-borne disease transmission		
Negative	16	(100)

### Surgical and Transplant Characteristics

Some donors received AVP (n = 9; 56%) within 24 h before cross-clamping (i.e., clamping of a major vessel to stop blood-flow towards the harvested organ) or inotropic medications during organ procurement (n = 11; 69%; Table 3).

**TABLE 3:** Surgical and transplant characteristics. Reported as n (%), unless otherwise stated.

Surgical and transplant characteristics	Patients (n = 16)	
Previous transplant of same organ type		
Yes	1	(6.3)
No	15	(94)
Multiple-VCA-transplant		
Yes	1	(6.3)
No	15	(94)
Extra allograft used		
Yes	8	(50)
No	6	(38)
Unknown	2	(13)
Warm ischemia time [min], median (IQR)	2.0	(0.0–75)
Cold ischemia time [min], median (IQR)	150	(90–195)
Distance donor-hospital to transplant center [nM], median (IQR)	19	(0.0–79)
Donor procurement with inotropic medication		
Yes	11	(69)
No	5	(31)
Donor pre-cross clamp administration of arginine vasopressin		
Yes	9	(56)
No	7	(44)
Donor pre-cross clamp administration of insulin		
Yes	6	(38)
No	10	(63)
Donor protein in urine		
Yes	6	(38)
No	10	(63)
Recipient pre-transplant blood transfusion		
Yes	12	(75)
No	2	(13)
Unknown	2	(13)
Recipient Coagulopathies		
Yes	1	(6.3)
No	13	(81)
Unknown	2	(13)
Recipient pre-transplant life support		
No	15	(94)
Unknown	1	(6.3)
Recipient Other risk factors		
Yes	4	(25)
No	10	(63)
Unknown	2	(13)
Recipient tolerance induction technique		
No	14	(88)
Unknown	2	(13)
Skin Type		
Type I (scores 0–6) Pale white; blond/red hair; blue eyes; freckles; always burns, never tans	1	(6.3)
Type II (scores 7–13) White; fair; blond/red hair; blue/green/hazel eyes; usually burns, tans minimally	2	(13)
Type III (scores 14–20) Cream white; fair, any hair/eye color; quite common; sometimes mild burn, tans uniformly	4	(25)
Type V (scores 28–34) Dark brown; Middle Eastern skin types; Very rarely burns, tans very easily	1	(6.3)
Unknown	8	(50)
UNOS transplant region		
Region 1	10	(63)
Region 2	1	(6.3)
Region 7	1	(6.3)
Region 9	1	(6.3)
Region 10	3	(19)
UNOS listing region		
Region 1	4	(25)
Region 7	1	(6.3)
Region 9	1	(6.3)
Region 10	2	(13)
Unknown	8	(50)
Allocation type		
Local	14	(88)
Regional	1	(6.3)
National	1	(6.3)
Year of transplant		
2008	1	(6.3)
2009	1	(6.3)
2011	3	(19)
2012	1	(6.3)
2013	1	(6.3)
2014	3	(19)
2015	1	(6.3)
2016	1	(6.3)
2017	1	(6.3)
2018	1	(6.3)
2019	1	(6.3)
2020	1	(6.3)

### Outcomes in fVCA Transplant Recipients

Transplant recipients experienced a median of 5 (IQR 0.0–10) acute rejection episodes. The longer the recipient survived (p = 0.006), the more acute rejections were observed. Furthermore, A1 donor blood group (p = 0.047), and the use of inotropic medication during organ procurement (p = 0.033) correlated with increased frequency of acute rejection episodes. On the other hand, higher pre-transplant serum creatinine levels (p = 0.020), elevated hemoglobin A1c at discharge (p = 0.049), and better GH scores (p = 0.016) were linked to fewer acute rejection episodes (Tables 4–7).

**TABLE 4:** Postoperative outcomes in facial VCA transplant recipients. Reported as n (%), unless otherwise stated.

Outcomes	Patients (n = 16)	
Number of acute rejection episodes, median (IQR)	5	(0.0–10)
Number of hospitalizations, mean ± SD	2.4	±1.8
Any complication	7	(44)
New-onset diabetes mellitus	2	(13)
Metabolic complication	2	(13)
Infectious complication	7	(44)
Other complication	3	(19)

**TABLE 5:** Numerical risk-associated factors for complications.

	Number of acute rejection episodes		Number of hospitalizations		Any complication	
	Spearman’s Coefficient	p-value	Spearman’s Coefficient	p-value	Spearman’s Coefficient	p-value
Recipient characteristics						
Age						
At transplantation	−0.27	0.31	−0.26	0.34	−0.42	0.10
At listing	−0.57	0.14	−0.65	0.081	−0.62	0.10
BMI						
At transplantation	0.034	0.90	0.24	0.39	−0.16	0.58
At registration	0.63	0.13	0.71	0.074	0.72	0.067
Weight						
At transplantation	−0.032	0.91	−0.018	0.95	−0.22	0.44
At listing	0.56	0.15	0.38	0.35	0.45	0.26
At registration	0.56	0.15	0.38	0.35	0.45	0.26
Height						
At transplantation	0.14	0.61	−0.44	0.10	0.0	>0.99
At registration	0.54	0.17	0.32	0.45	0.40	0.33
CPRA score						
At transplantation	0.28	0.32	0.10	0.72	−0.07	0.82
At listing	−0.14	0.74	−0.23	0.58	−0.17	0.69
Days on liver waiting list	−0.17	0.70	−0.18	0.66	−0.17	0.69
Survival time	0.68	**0.006**	0.82	**<0.001**	0.50	0.061
Laboratory values						
Serum creatinine						
Pre-transplant	−0.59	**0.020**	−0.49	0.065	−0.37	0.17
Discharge	−0.34	0.23	−0.57	**0.033**	−0.12	0.67
Hemoglobin A1c						
Pre-transplant	−0.62	0.075	0.0	>0.99	0.19	0.62
Discharge	−0.71	**0.049**	−0.43	0.29	−0.13	0.76
Warm ischemia time	−0.53	0.14	−0.16	0.68	−0.23	0.56
Cold ischemia time	−0.015	0.96	−0.013	0.97	−0.31	0.30
Donor characteristics						
Age	0.087	0.75	0.14	0.59	−0.26	0.33
BMI	0.21	0.43	0.41	0.11	0.31	0.24
Weight	−0.033	0.90	0.077	0.78	0.12	0.65
Height	−0.32	0.22	−0.39	0.13	−0.082	0.76
Surgical characteristics						
Distance donor-hospital to transplant center	−0.15	0.58	−0.16	0.55	−0.014	0.96
Computed donor antigens						
DA1	0.14	0.61	0.11	0.70	0.39	0.13
DA2	0.43	0.10	0.025	0.93	0.30	0.25
DB1	0.32	0.23	0.082	0.76	0.13	0.65
DB2	0.40	0.12	0.45	0.080	0.12	0.65
DDR1	0.26	0.33	−0.24	0.38	0.0	>0.99
DDR2	−0.24	0.36	−0.016	0.95	−0.14	0.60
Evaluated Scores						
Physical Functioning (PF) score	−0.58	0.23	0.088	0.87	−0.29	0.57
Role-Physical (RP) score	−0.64	0.17	−0.12	0.82	−0.49	0.33
Bodily Pain (BP) score	0.37	0.47	0.81	0.053	0.59	0.21
General Health (GH) score	−0.89	**0.016**	−0.76	0.079	−0.89	**0.017**
Vitality (VT) score	−0.33	0.52	0.0	>0.99	−0.29	0.57
Social Functioning (SF)	−0.091	0.86	0.24	0.65	0.10	0.85
Role-Emotional (RE)	0.48	0.33	0.28	0.59	0.31	0.55
Mental Health (MH) score	−0.46	0.44	−0.76	0.13	−0.59	0.29

Statistically significant p-values are highlighted in bold.

**TABLE 6:** Categorical risk-associated factors for complications.

	Number of acute rejection episodes		Number of hospitalizations		Any complication	
	H-statistic	p-value	H-statistic	p-value	H-statistic	p-value
Recipient characteristics						
Gender	0.75	0.39	2.4	0.12	0.73	0.39
Primary diagnosis	0.014	0.91	0.0040	0.95	0.67	0.41
AB0	0.41	0.52	0.57	0.45	0.80	0.37
A1 antigen	0.067	0.80	0.051	0.82	0.0	>0.99
DR1 antigen	1.0	0.32	1.5	0.22	1.0	0.32
DR2 antigen	2.7	0.10	2.4	0.12	3.0	0.083
CMV status	0.17	0.68	1.3	0.25	1.5	0.22
Pre-transplant hospitalisation within 90 days	0.029	0.86	0.0070	0.93	0.010	0.92
Pre-transplant transfusion	3.4	0.064	2.2	0.14	2.2	0.14
Donor characteristics						
Gender	0.75	0.39	2.4	0.12	0.73	0.39
AB0	6.1	**0.047**	4.8	0.090	4.3	0.12
EBV (VCA) (IgG) status	1.7	0.20	0.059	0.81	0.034	0.85
Tattoos	1.3	0.25	0.21	0.64	0.039	0.84
Protein in donor urine	0.51	0.47	0.0	>0.99	1.9	0.17
ECD	0.0040	0.95	0.034	0.85	3.9	**0.049**
General immunological characteristics						
HLA mismatch level	2.39	0.30	0.13	0.94	0.17	0.92
A locus mismatch level	2.02	0.37	3.22	0.20	2.02	0.36
DR locus mismatch level	2.86	0.09	0.00	1.00	0.47	0.50
Surgical characteristics						
Year of transplant	0.20	0.66	2.4	0.12	2.5	0.11
UNOS transplant region	2.4	0.13	4.0	**0.047**	4.2	**0.040**
UNOS listing region	2.2	0.14	3.9	**0.049**	5.0	**0.025**
Use of extra allograft	2.5	0.12	2.5	0.11	1.1	0.30
Skin type	0.0	>0.99	3.5	0.060	2.5	0.11
Donor pre-transplant administration of arginine vasopressin	2.4	0.12	4.4	**0.035**	0.85	0.36
Donor pre-transplant administration of insulin	0.20	0.66	1.9	0.17	0.14	0.71
Donor procurement with inotropic medication	4.5	**0.033**	0.16	0.69	1.6	0.21

Statistically significant p-values are highlighted in bold.

**TABLE 7:** Categorical results and post-hoc Dunn’s test with Bonferroni correction of significant categorical risk-associated factors.

	p-value	Category	Median
Number of acute rejection episodes			
Donor procurement with inotropic medication			
Yes vs. No	**0.033**	Yes	8.0
		No	0.0
Donor ABO			
A vs. A1	0.368*	A	4.0
A vs. 0	1.0*	A1	16.0
A1 vs. 0	0.047*	0	3.0
Number of hospitalizations			
Donor pre-transplant administration of arginine vasopressin			
Yes vs. No	**0.035**	Yes	1.0
		No	4.0
UNOS transplant region			
1 vs. 10	**0.047**	1	2.5
		10	5.0
UNOS listing region			
1 vs. 10	**0.049**	1	0.0
		10	4.0
Any complication			
Donor ECD			
Yes vs. No	**0.049**	Yes	1.0
		No	0.0
UNOS transplant region			
1 vs. 10	**0.040**	1	0.0
		10	1.0
UNOS listing region			
1 vs. 10	**0.025**	1	0.0
		10	1.0

^*^corrected p-values.

Statistically significant p-values are highlighted in bold.

On average, patients were hospitalized 2.4 ± 1.8 times during the follow-up period. The longer the recipient survived (p = 0.001), the more hospitalizations were observed. Conversely, higher serum creatinine levels at discharge (p = 0.033), and donor administration of AVP before cross-clamping (p = 0.035) were associated with fewer hospitalizations post-transplant (Tables 4–6).

Complications occurred in seven patients (44%), with specific complications including new-onset diabetes mellitus (n = 2; 13%), metabolic issues (n = 2; 13%), infectious complications (n = 7; 44%), and other types of complications (n = 3; 19%). Positive ECD status was associated with higher complication rates (p = 0.049), while a higher GH score was linked to fewer occurrences of complications (p = 0.017; Tables 4–6).

## Discussion

Big databases present a tool for tracking outcomes, identifying associated factors, and improving patient care. fVCA patients are particularly vulnerable due to multiple factors, such as severity of initial trauma, surgical complexity, and the need for life-long immunosuppression. Thus, we analyzed 16 cases of fVCA from the OPTN database to identify potential risk factors correlating with postoperative complications.

Acute rejection is one of the most common complications in VCA transplantation occurring more frequently than in solid organ transplants (SOT) [14]. In our study, we found a positive correlation between the use of inotropic medication during donor organ procurement and the frequency of postoperative acute rejection episodes.

From a broader perspective, our results align with studies in SOT. For example, Nixon et al. and D’Ancona et al. showed that high-dose inotrope donor support had a higher tendency for early post-transplant complications and was the major determinant for primary graft failure after heart transplantation [15, 16]. While Blitzer et al. concluded that donor inotropic medication did not impact short-term heart transplant recipient survival, administration of even one inotrope was associated with increased 1-year mortality by 14% [17]. This lends support to the hypothesis that donor hemodynamic maintenance with inotropes influences outcomes of organ transplantation, such as postoperative acute rejection episodes. This might be due to the wide-spread ischemic consequences of vasoconstriction [18, 19]. To overcome this obstacle, Westphal et al. suggested the additional use of hormone replacement therapy (i.e., vasopressin, thyroid hormones, and corticosteroids) to improve the hemodynamics of deceased donors, ultimately decreasing the need for inotropic medication.

Overall, reducing the need for inotrope administration in potential donors during organ procurement might improve post-transplant outcomes of fVCA, and decrease rates of acute rejection episodes. However, future research is warranted to determine the optimal drug regime for hemodynamic management in potential donors.

In our study, we also found a direct correlation between donor administration of AVP during organ procurement and decreased frequency of postoperative hospitalizations after fVCA.

Broadly speaking, these findings echo previous studies that report improved medical outcomes after donor administration of AVP during solid organ procurement [20–22]. For instance, in their analysis of lung transplants using the OPTN database, Callahan et al. observed a notable rise in the number of successfully procured organs and enhanced preservation of transplanted lung function when donors were administered AVP. They propose that AVP exerts a catecholamine-sparing effect, reducing the need for inotropes in cases of brain-death-induced cardiovascular collapse, minimizing inflammatory mediator release, and decreasing reliance on crystalloid supportive therapy [21].

Additional evidence highlights the beneficial effects of AVP on blood pressure, vascular tone, and the need for inotropic medication. Pennefather et al. demonstrated that administering AVP to brain-dead donors significantly reduced plasma hyperosmolality and inotrope requirements, while improving blood pressure and hemodynamic stability. They noted that low-dose AVP infusion allows for a reduction in inotropic support without causing adverse hemodynamic effects, thereby mitigating the detrimental impact of catecholamines on transplant outcomes. Furthermore, AVP administration was associated with a decrease in postoperative hospitalizations [23].

Nakagawa et al. further reported that AVP contributed to maintaining hemodynamic stability and fluid homeostasis in deceased organ donors, ultimately improving both the quality and quantity of transplanted organs and enhancing post-transplant organ function [24]. However, Pennefather et al. also cautioned that insufficient AVP dosing carries a significant risk of cardiovascular overstimulation, potentially leading to organ damage or a decline in organ quality [23].

Our findings align with previous research demonstrating that donor administration of AVP during organ procurement is associated with improved postoperative outcomes, including reduced hospitalizations, enhanced hemodynamic stability, and better organ preservation. These results further support the role of AVP in optimizing donor management strategies to improve transplant success.

The concept of ECD encompasses organ donors who possess one or more characteristics that may adversely affect transplant outcomes—such as advanced age, a history of smoking, or pre-existing comorbidities like diabetes—yet are utilized in order to address the persistent organ shortage in SOT [25, 26]. While multiple studies have reported mixed findings regarding the impact of ECD on transplant complications and clinical outcomes, their use is steadily increasing due to the urgent need for grafts. In our study, we found that the use of ECD donor organs in fVCA correlated with higher rates of postoperative complications, aligning with previously documented trends in liver and kidney transplantation.

For instance, ECD liver grafts have been associated with an increased incidence of severe surgical complications (60% vs. 45%), graft loss (14% vs. 8%), and mortality (14% vs. 4%), as identified by Pagano et al. [27] In addition, ECD liver recipients are at higher risk for primary nonfunction, biliary complications, and graft-related fatalities (approximately 10%), with a further 10% of patients requiring re-listing for a second transplant within 1 year of their initial procedure [28–30]. However, ECD kidney transplants present a more nuanced picture: although Fellmann et al. observed no significant increase in recipient postoperative complication rates *per se*, they did identify a heightened risk of delayed graft function and diminished graft survival—factors attributable to ischemia-reperfusion injury—while overall recipient survival remained unaffected [31, 32]. Importantly, they argued that many observed complications in SOT recipients derive more from pre-existing comorbidities of the recipient such as diabetes, cardiovascular disease, and the necessity for anticoagulation therapy than from ECD status alone.

Together, these findings underscore the importance of meticulous patient selection, thorough preoperative risk stratification, and comprehensive informed consent—particularly when considering the use of ECD grafts in VCA. Although employing ECDs may bolster the donor pool and mitigate organ shortages, vigilance is warranted to balance the potential for increased complication rates with the life-enhancing benefits that transplantation can provide.

## Limitations

To the best of our knowledge, this study is the first to analyze acute complication and hospitalization rates, as well as the occurrence of complications in fVCA cases using multi-center data collected over more than a decade. However, it is essential to interpret these findings in light of the study’s limitations.

First, the statistical analyses used in this study revealed correlations rather than causal relationships, meaning that the underlying causal mechanisms remain unclear. Additionally, our data was extracted from the OPTN database, which provided only 16 fVCA cases with follow-ups, thereby limiting the sample size.

The retrospective nature of the study also introduces the potential risk of bias and confounding factors. Inconsistencies in data collection across centers, due to the varying expertise and subjectivity of database contributors, present a challenge for intra- and interinstitutional data comparisons. This may impair the of the dataset [33].

Furthermore, because the OPTN is a national U.S. database, the study is inherently limited by its focus on the U.S. healthcare system. As a result, ethical and racial disparities in VCA donation and transplantation may not be fully addressed, limiting the generalizability of the findings [34]. The lack of standardized criteria for diagnosing and validating episodes of acute rejection in the OPTN database further complicates the interpretation of rejection-related outcomes. More specifically, the OPTN database only records the date of an acute rejection episode but lacks details regarding the diagnostic methods or validation process.

Despite these limitations, we believe that this study makes a valuable contribution to the field by offering insights into potential risk factors and new strategies for fVCA transplantation. The findings provide a foundation for future research, which may expand on these results to clarify underlying mechanisms and improve patient outcomes.

## Conclusion

In summary, this study identified novel factors that influence postoperative outcomes in fVCA. We found that inotropic medication use during donor procurement was correlated with higher rates of acute rejection, consistent with trends seen in SOT. In contrast, the use of AVP was associated with fewer postoperative hospitalizations, which may improve donor stability and transplant outcomes. Our findings also highlighted increased complications in ECD-based fVCA, reinforcing the need for careful patient selection and preoperative evaluation. While the study has limitations, including small sample size and retrospective design, this line of research may unlock untapped potential for improving fVCA management. Future prospective studies are needed to confirm these findings and optimize the perioperative donor and recipient management for improved transplant success.

## Data Availability

The original contributions presented in the study are included in the article/supplementary material, further inquiries can be directed to the corresponding author.

## References

[1] Kauke-Navarro M Knoedler L Deniz C Knoedler S Safi A-F. Early Outcomes and Risk Factors for Complications After Facial Alloplastic Implant Surgery – An ACS-NSQIP Study. J Plast Reconstr and Aesthet Surg (2024) 90:209–14. 10.1016/j.bjps.2024.02.021 38387417

[2] Kauke M Safi AF Zhegibe A Haug V Kollar B Nelms L Mucosa and Rejection in Facial Vascularized Composite Allotransplantation: A Systematic Review. Transplantation (2020) 104(12):2616–24. 10.1097/tp.0000000000003171 32053572

[3] Kauke M Panayi AC Safi AF Haug V Perry B Kollar B Full Facial Retransplantation in a Female Patient—Technical, Immunologic, and Clinical Considerations. Am J Transplant (2021) 21(10):3472–80. 10.1111/ajt.16696 34033210

[4] Kauke-Navarro M Knoedler S Panayi AC Knoedler L Haller B Parikh N Correlation Between Facial Vascularized Composite Allotransplantation Rejection and Laboratory Markers: Insights From a Retrospective Study of Eight Patients. J Plast Reconstr and Aesthet Surg (2023) 83:155–64. 10.1016/j.bjps.2023.04.050 37276734

[5] Kauke-Navarro M Knoedler L Knoedler S Diatta F Huelsboemer L Stoegner VA Ensuring Racial and Ethnic Inclusivity in Facial Vascularized Composite Allotransplantation. Plast Reconstr Surg Glob Open (2023) 11(8):e5178. 10.1097/gox.0000000000005178 37577247 PMC10419650

[6] Sarhane KA Tuffaha SH Broyles JM Ibrahim AE Khalifian S Baltodano P A Critical Analysis of Rejection in Vascularized Composite Allotransplantation: Clinical, Cellular and Molecular Aspects, Current Challenges, and Novel Concepts. Front Immunol (2013) 4:406. 10.3389/fimmu.2013.00406 24324470 PMC3839257

[7] Ravindra KV Xu H Bozulic LD Song DD Ildstad ST. The Need for Inducing Tolerance in Vascularized Composite Allotransplantation. Clin Dev Immunol (2012) 2012:438078. 10.1155/2012/438078 23251216 PMC3509522

[8] Kaufman CL Marvin MR Chilton PM Hoying JB Williams SK Tien H Immunobiology in VCA. Transpl Int (2016) 29(6):644–54. 10.1111/tri.12764 26924305

[9] Etra JW Raimondi G Brandacher G. Mechanisms of Rejection in Vascular Composite Allotransplantation. Curr Opin Organ Transpl (2018) 23(1):28–33. 10.1097/mot.0000000000000490 29189293

[10] Siemionow M Ozturk C. Face Transplantation: Outcomes, Concerns, Controversies, and Future Directions. J Craniofac Surg (2012) 23(1):254–9. 10.1097/SCS.0b013e318241b920 22337420

[11] Fischer S Kueckelhaus M Pauzenberger R Bueno EM Pomahac B. Functional Outcomes of Face Transplantation. Am J Transpl (2015) 15(1):220–33. 10.1111/ajt.12956 25359281

[12] Hadjiandreou M Pafitanis G Butler PM. Outcomes in Facial Transplantation - A Systematic Review. Br J Oral Maxillofac Surg (2024) 62(5):404–14. 10.1016/j.bjoms.2024.02.008 38637216

[13] Hernandez JA Testa G Naga HI Pogson KB Miller JM Booker SE OPTN/SRTR 2021 Annual Data Report: Vascularized Composite Allograft. Am J Transpl (2023) 23(2 Suppl. 1):S523–s545. 10.1016/j.ajt.2023.02.012 37132342

[14] Fischer S Lian CG Kueckelhaus M Strom TB Edelman ER Clark RA Acute Rejection in Vascularized Composite Allotransplantation. Curr Opin Organ Transpl (2014) 19(6):531–44. 10.1097/MOT.0000000000000140 25333831

[15] Nixon JL Kfoury AG Brunisholz K Horne BD Myrick C Miller DV Impact of High-Dose Inotropic Donor Support on Early Myocardial Necrosis and Outcomes in Cardiac Transplantation. Clin Transpl (2012) 26(2):322–7. 10.1111/j.1399-0012.2011.01504.x 21981698

[16] D'Ancona G Santise G Falletta C Pirone F Sciacca S Turrisi M Primary Graft Failure After Heart Transplantation: The Importance of Donor Pharmacological Management. Transpl Proc (2010) 42(3):710–2. 10.1016/j.transproceed.2010.03.027 20430153

[17] Blitzer D Baran DA Lirette S Copeland JG Copeland H. Does Donor Treatment With Inotropes and/or Vasopressors Impact Post-Transplant Outcomes? Clin Transpl (2023) 37(4):e14912. 10.1111/ctr.14912 36650699

[18] Rodrigues Sde L. Profile of Effective Donors from Organ and Tissue Procurement Services. Rev Bras Ter Intensiva (2014) 26(1):21–7. 10.5935/0103-507x.20140004 24770685 PMC4031888

[19] Ditonno P Impedovo SV Palazzo S Bettocchi C Gesualdo L Grandaliano G Effects of Ischemia-Reperfusion Injury in Kidney Transplantation: Risk Factors and Early and Long-Term Outcomes in a Single Center. Transpl Proc (2013) 45(7):2641–4. 10.1016/j.transproceed.2013.07.025 24034012

[20] Benck U Gottmann U Hoeger S Lammert A Rose D Boesebeck D Donor Desmopressin Is Associated With Superior Graft Survival After Kidney Transplantation. Transplantation (2011) 92(11):1252–8. 10.1097/TP.0b013e318236cd4c 22067309

[21] Callahan DS Neville A Bricker S Kim D Putnam B Bongard F The Effect of Arginine Vasopressin on Organ Donor Procurement and Lung Function. J Surg Res (2014) 186(1):452–7. 10.1016/j.jss.2013.09.028 24176209

[22] Plurad DS Bricker S Falor A Neville A Bongard F Putnam B. Donor Hormone and Vasopressor Therapy: Closing the Gap in a Transplant Organ Shortage. J Trauma Acute Care Surg (2012) 73(3):689–94. 10.1097/TA.0b013e318250b122 22710780

[23] Pennefather SH Bullock RE Mantle D Dark JH. Use of Low Dose Arginine Vasopressin to Support Brain-Dead Organ Donors. Transplantation (1995) 59(1):58–62. 10.1097/00007890-199501150-00011 Accessed December 10, 2024. 7839429

[24] Nakagawa K Tang JF. Physiologic Response of Human Brain Death and the Use of Vasopressin for Successful Organ Transplantation. J Clin Anesth (2011) 23(2):145–8. 10.1016/j.jclinane.2009.12.015 21377081

[25] Berenguer M. Risk of Extended Criteria Donors in Hepatitis C Virus-Positive Recipients. Liver Transpl (2008) 14(Suppl. 2):S45–50. 10.1002/lt.21617 18825715

[26] Mulligan MJ Sanchez PG Evans CF Wang Y Kon ZN Rajagopal K The Use of Extended Criteria Donors Decreases One-Year Survival in High-Risk Lung Recipients: A Review of the United Network of Organ Sharing Database. J Thorac Cardiovasc Surg (2016) 152(3):891–8. 10.1016/j.jtcvs.2016.03.096 27234027

[27] Pagano D Barbàra M Seidita A Cintorino D di Francesco F Petridis I Impact of Extended-Criteria Donor Liver Grafts on Benchmark Metrics of Clinical Outcome After Liver Transplantation: A Single Center Experience. Transplant Proc (2020) 52(5):1588–92. 10.1016/j.transproceed.2020.02.050 32222388

[28] Manzarbeitia CY Ortiz JA Jeon H Rothstein KD Martinez O Araya VR Long-Term Outcome of Controlled, Non—Heart-Beating Donor Liver Transplantation. Transplantation (2004) 78(2):211–5. 10.1097/01.tp.0000128327.95311.e3 15280680

[29] Fujita S Mizuno S Fujikawa T Reed AI Kim RD Howard RJ Liver Transplantation From Donation After Cardiac Death: A Single Center Experience. Transplantation (2007) 84(1):46–9. 10.1097/01.tp.0000267424.88023.7b 17627236

[30] Jay CL Lyuksemburg V Ladner DP Wang E Caicedo JC Holl JL Ischemic Cholangiopathy After Controlled Donation After Cardiac Death Liver Transplantation: A Meta-Analysis. Ann Surg (2011) 253(2):259–64. 10.1097/SLA.0b013e318204e658 21245668

[31] Domagala P Kwiatkowski A Wszola M Czerwinski J Cybula K Trzebicki J Complications of Transplantation of Kidneys From Expanded-Criteria Donors. Transpl Proc (2009) 41(8):2970–1. 10.1016/j.transproceed.2009.07.085 19857652

[32] Fellmann M Balssa L Clément E Frey P Frontczak A Bernardini S Postoperative Complications and Long-Term Outcomes of Transplantation With Expended Criteria Donors Transplants. Prog Urol (2020) 30(12):655–62. 10.1016/j.purol.2020.04.019 32814659

[33] Norgaard M Ehrenstein V Vandenbroucke JP. Confounding in Observational Studies Based on Large Health Care Databases: Problems and Potential Solutions - A Primer for the Clinician. Clin Epidemiol (2017) 9:185–93. 10.2147/CLEP.S129879 28405173 PMC5378455

[34] Kimberly LL Ramly EP Alfonso AR Diep GK Berman ZP Rodriguez ED. Equity in Access to Facial Transplantation. J Med Ethics (2020) 47:e10. 10.1136/medethics-2020-106129 33060187

